# Transcriptome analysis in oak uncovers a strong impact of endogenous rhythmic growth on the interaction with plant-parasitic nematodes

**DOI:** 10.1186/s12864-016-2992-8

**Published:** 2016-08-12

**Authors:** Hazel R. Maboreke, Lasse Feldhahn, Markus Bönn, Mika T. Tarkka, Francois Buscot, Sylvie Herrmann, Ralph Menzel, Liliane Ruess

**Affiliations:** 1 Institute of Biology, Ecology Group, Humboldt-Universität zu Berlin, Philippstr. 13, 10115 Berlin, Germany; 2 Department of Soil Ecology, UFZ – Helmholtz Centre for Environmental Research, Theodor-Lieser-Str. 4, 06120 Halle/Saale, Germany; 3 Department of Community Ecology, UFZ – Helmholtz Centre for Environmental Research, Theodor-Lieser-Str. 4, 06120 Halle/Saale, Germany; 4 German Centre for Integrative Biodiversity Research (iDiv) Halle-Jena-Leipzig, Deutscher Platz 5e, 04103 Leipzig, Germany

**Keywords:** Plant-parasitic nematode, Oak rhythmic growth, Ectomycorrhiza, Systemic response, Defence, Transcriptomic profile

## Abstract

**Background:**

Pedunculate oak (*Quercus robur* L.), an important forest tree in temperate ecosystems, displays an endogenous rhythmic growth pattern, characterized by alternating shoot and root growth flushes paralleled by oscillations in carbon allocation to below- and aboveground tissues. However, these common plant traits so far have largely been neglected as a determining factor for the outcome of plant biotic interactions. This study investigates the response of oak to migratory root-parasitic nematodes in relation to rhythmic growth, and how this plant-nematode interaction is modulated by an ectomycorrhizal symbiont. Oaks roots were inoculated with the nematode *Pratylenchus penetrans* solely and in combination with the fungus *Piloderma croceum*, and the systemic impact on oak plants was assessed by RNA transcriptomic profiles in leaves.

**Results:**

The response of oaks to the plant-parasitic nematode was strongest during shoot flush, with a 16-fold increase in the number of differentially expressed genes as compared to root flush. Multi-layered defence mechanisms were induced at shoot flush, comprising upregulation of reactive oxygen species formation, hormone signalling (e.g. jasmonic acid synthesis), and proteins involved in the shikimate pathway. In contrast during root flush production of glycerolipids involved in signalling cascades was repressed, suggesting that *P. penetrans* actively suppressed host defence. With the presence of the mycorrhizal symbiont, the gene expression pattern was vice versa with a distinctly stronger effect of *P. penetrans* at root flush, including attenuated defence, cell and carbon metabolism, likely a response to the enhanced carbon sink strength in roots induced by the presence of both, nematode and fungus. Meanwhile at shoot flush, when nutrients are retained in aboveground tissue, oak defence reactions, such as altered photosynthesis and sugar pathways, diminished.

**Conclusions:**

The results highlight that gene response patterns of plants to biotic interactions, both negative (i.e. plant-parasitic nematodes) and beneficial (i.e. mycorrhiza), are largely modulated by endogenous rhythmic growth, and that such plant traits should be considered as an important driver of these relationships in future studies.

**Electronic supplementary material:**

The online version of this article (doi:10.1186/s12864-016-2992-8) contains supplementary material, which is available to authorized users.

## Background

Pedunculate oak (*Quercus robur* L.) plays an important ecological role by supporting a high biodiversity of above and below ground living animals that interact with the host tree and with each other [1, 2]. The vegetative development of oak trees is characterized by an endogenous rhythmic growth with alternation of shoot flush (SF) and root flush (RF), paralleled by oscillations in photo-assimilate allocation to either emerging buds or growing fine roots [3, 4]. Comparably, Angay et al. [5] showed that the rhythmic growth of *Q. robur* resulted in high amounts of non-structural carbohydrates in roots during RF and low quantities during SF. Moreover, the rhythmic growth strongly relates to fluctuations of transcriptome patterns in both below- and aboveground tissues of oaks [4].

Like many temperate forest trees oak forms a symbiotic relationship with ectomycorrhizal fungi to enhance nutrient acquisition [6]. Several studies have reported an extensive re-programming of the oak transcriptome during both the pre-symbiotic and mature symbiotic states with *Piloderma croceum* as a mycorrhiza partner [7–9]. In contrast, a lack of knowledge exists on the mechanism by which oak trees integrate signals induced by belowground herbivores into their endogenous rhythmic growth at the gene expression level and if these interactions are affected by the presence of a mycorrhizal symbiont.

Plant-parasitic nematodes are responsible for important damages to crops, which on a global scale have been estimated as financial losses of $ 80 - 118 billion annually [10]. Over the last decade molecular tools such as microarrays and RNA-Seq analyses have allowed disentanglement of these plant-nematode interactions at the transcriptomic level [11, 12]. Plant-parasitic nematodes induce changes in host plant gene expression patterns at local level and also distant tissues via systemic signalling [13, 14]. These include manipulation of host plant cell physiology, cell morphogenesis, hormone balance as well as suppressing plant’s stress and defence responses [15–19]. While studies on localized responses identify host feedback directly regulated by nematodes, investigations on distant and systemic responses provide a broader understanding on plant health in relation to plant-nematode interactions [20].

Apart from agricultural crops, nematodes also impair plant performance in natural ecosystems including forests [21], e.g. the genus *Pratylenchus* was shown to hamper tree nutrient acquisition via ectomycorrhiza fungi [22, 23]. However, localized and systemic responses of plants to nematode infection have been in the focus of investigations on agricultural crops [24–26], whereas knowledge in forest trees remains scarce. To support these kinds of studies, Tarkka et al. [9] generated a reference library for differential gene expression of pedunculate oak during series of beneficial and detrimental below and above ground biotic interactions (OakContigDF159.1) in the frame of the research consortium TrophinOak [27]. The OakContigDF159.1 assembly was based on a set of 18 cDNA libraries from oak roots and leaves interacting with different organisms including the nematode *Pratylenchus penetrans*. This transcriptome library comprises of more than 60,000 contigs allowing for the analysis of differential gene expression in experiments on interspecific interactions.

The aim of the present study was to unravel the systemic transcription changes expressed in oak leaves in response to root-herbivory by the migratory endoparasitic nematode *P. penetrans*, and analyse how this plant-nematode interaction is altered by oak’s endogenous rhythmic growth in the presence or absence of a mycorrhizal partner *P. croceum*. This work was based on three hypotheses. Our first hypothesis states that *P. penetrans* induces genes associated with defence response and secondary metabolism in leaves, while our second hypothesis states that these *P. penetrans* induced plant responses vary according to the oak’s rhythmic growth phase. Our third hypothesis states that the interaction with *P. croceum* primes oaks against infection by pathogens, thereby altering the defence response to nematodes. The third hypothesis is based on general literature stating that ectomycorrhizal colonization of roots induces genes related to flavonoid biosynthesis and alterations in plant secretions both involved in plant-pathogen interaction [28, 29]. To address these hypotheses microcuttings of *Q. robur* were used as a miniaturized model in a soil based culture system to investigate systemic transcriptomic changes in leaves during SF and RF.

## Results

### Differential gene expression with oak biotic interactions

**Fig. 1:**
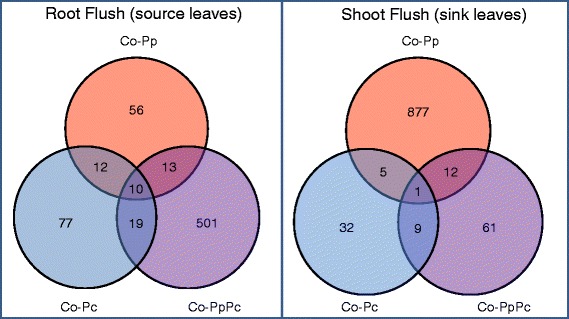
Venn diagram illustrating the numbers of significant differentially expressed contigs (DECs). Overlapping areas represent DECs common to different inoculation treatments. The figure compares the following pairs of oak microcuttings during root flush and shoot flush: Control versus *Pratylenchus penetrans* (Co-Pp), Control versus *Piloderma croceum* (Co-Pc) and Control versus sequential-inoculation of *P. penetrans* and *P. croceum* (Co-PpPc). FDR cut-off = 0.01

The infection of the oak microcuttings by *P. penetrans* was visually confirmed by microscopic observation. Pairwise comparative gene expression profiling of datasets from non-inoculated plants versus plants inoculated with *P. penetrans* (Pp) and plants inoculated with both *P. penetrans* and *P. croceum* (PpPc). The numbers of obtained differentially expressed contigs (DECs) are presented in Venn diagrams shown in Fig. 1 and tabulated in Table 1. The response of microcuttings to *P. penetrans* infection was greatly influenced by the plant’s growth stage demonstrated by a 16-fold increase in the number of DECs during SF compared to RF (Fig. 1). In contrast, the picture of the DECs was inverted with a 10-fold increase of DECs in response to the interaction with *P. penetrans* and *P. croceum* during RF compared to SF (Fig. 1). Irrespective of oak growth stage, there was a noticeably low overlap in co-expressed genes between biotic interactions, with common contigs not exceeding 13 and 12 during RF and SF, respectively (Fig. 1). The symbiotic interaction of oak with *P. croceum* was marginally mpacted by plant growth with a total number of 77 and 32 unique DECs during RF and SF, respectively. Cross comparisons of the DECs common in response to *P. penetrans* and co-inoculation of *P. penetrans* and *P. croceum* treatments between RF and SF revealed only two contigs (Additional file 1). These were a *cyclic nucleotide gated channel 1* involved in innate immunity that was upregulated in both treatments at both growth stages and *FUS*-*complementing gene 2* which is involved in mRNA processing and protein phosphorylation downregulated except for PpPc during SF.

**Table 1:** Pairwise comparison of differentially expressed genes

	Number of differentially expressed contigs		
	Co-Pp	Co-Pc	Co-PpPc
RF			
Total	91	118	543
Upregulated	46	63	371
Downregulated	45	55	172
SF			
Total	895	47	83
Upregulated	289	23	27
Downregulated	606	24	56

Table shows the numbers of differentially expressed contigs in oak microcutting leaves at different plant growth stages following pairwise comparisons. -Control versus *P. penetrans* (Co-Pp), Control versus *P. croceum* (Co-Pc), Control versus co-inoculation of *P. penetrans and P. croceum* (Co-PpPc), RF- root flush and SF- shoot flush. Significance of differential expression was determined using a threshold of Benjamini-Hochberg adjusted *P* < 0.01 as cut off

**Fig. 2:**
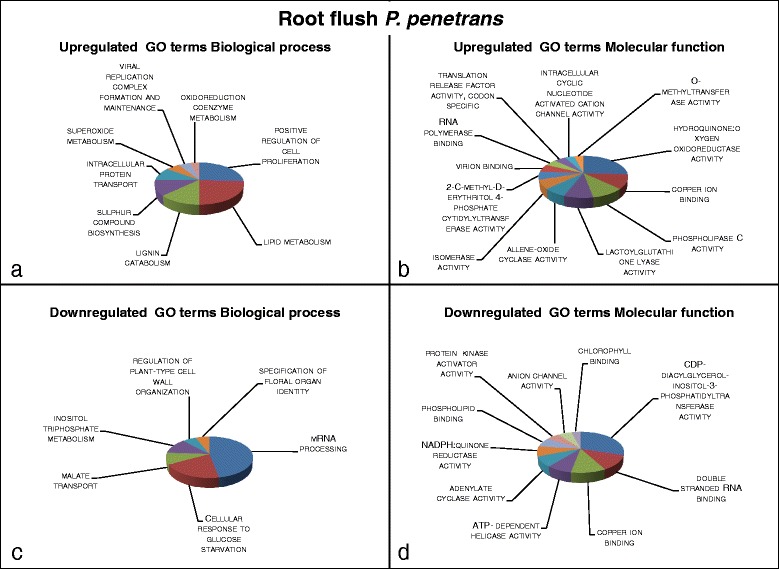
Visualization of summarized enriched GO terms expressed in systemic tissue of oak microcuttings in response to inoculation with *Pratylenchus penetrans* at root flush (RF); **a** GO terms with a biological process role enriched for 24 upregulated contigs; **b** GO terms with a molecular function role enriched for 16 upregulated contigs; **c** GO terms with a biological process role enriched for 25 downregulated contigs; **d** GO terms with a molecular function role enriched for 24 downregulated contigs

**Fig. 3:**
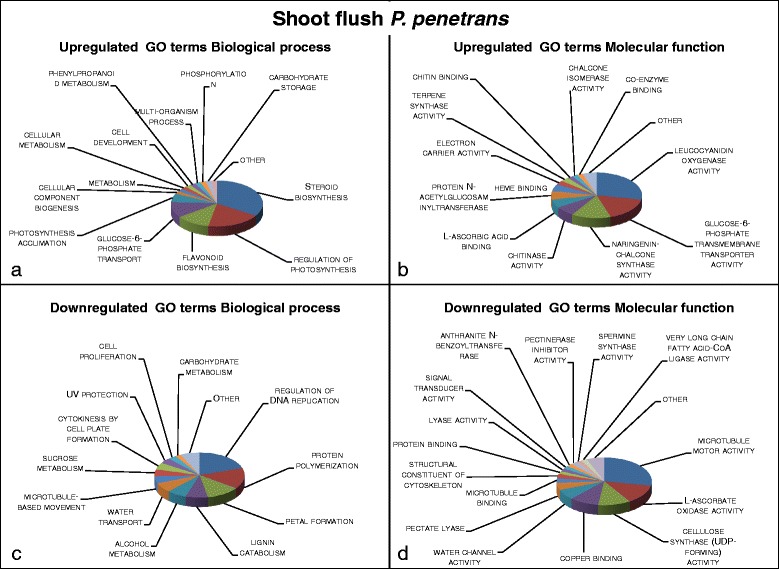
Visualization of summarized enriched GO terms expressed in systemic tissue of oak microcuttings in response to inoculation with *Pratylenchus penetrans* at shoot flush (SF); **a** GO terms with a biological process role enriched for 68 upregulated contigs; **b** GO terms with a molecular function role enriched for 60 upregulated contigs; **c** GO terms with a biological process role enriched for 178 downregulated contigs; **d** GO terms with a molecular function role enriched for 102 downregulated contigs

**Fig. 4:**
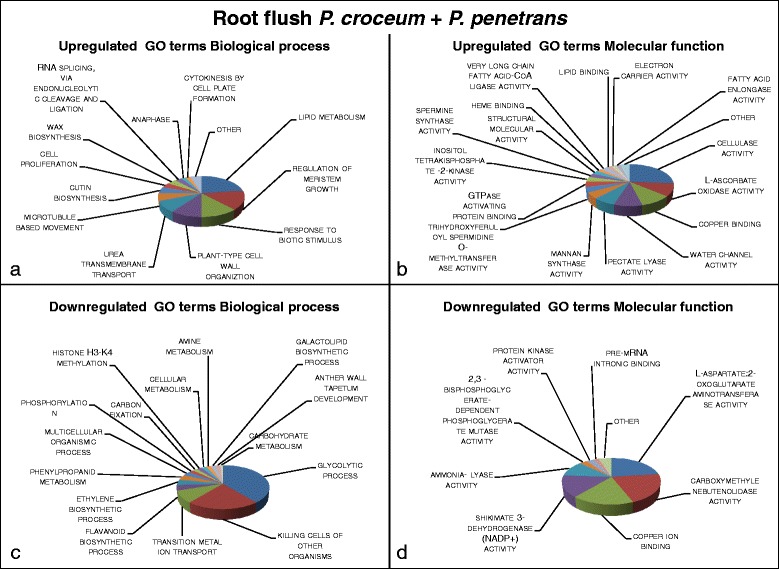
Visualization of summarized enriched GO terms expressed in systemic tissue of oak microcuttings in response to the co-inoculation of *Pratylenchus penetrans* and *Piloderma croceum* at root flush (RF); **a** GO terms with a biological process role enriched for 99 upregulated contigs; **b** GO terms with a molecular function role enriched for 61 upregulated contigs; **c** GO terms with a biological process role enriched for 68 downregulated contigs; **d** GO terms with a molecular function role enriched for 71 downregulated contigs

**Fig. 5:**
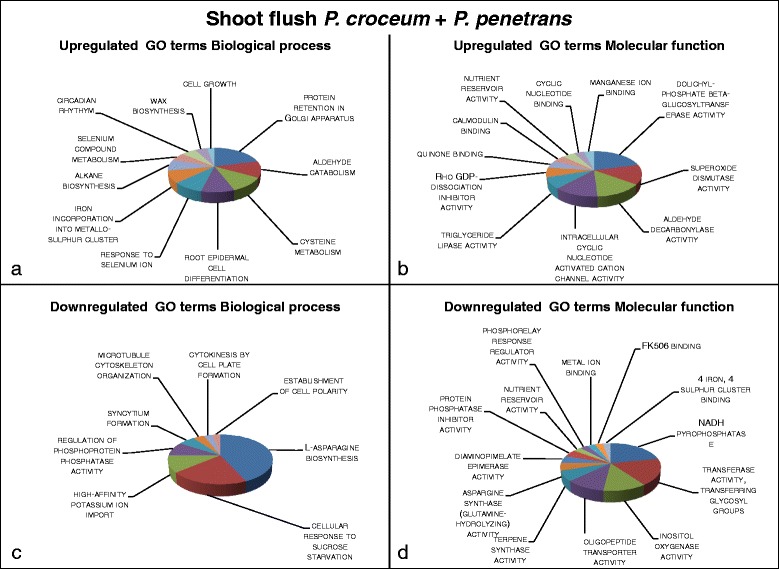
Visualization of summarized enriched Go terms expressed in systemic tissue of oak microcuttings in response to the co-inoculation of *Pratylenchus penetrans* and *Piloderma croceum* at shoot flush (SF); **a** GO terms with a biological process role enriched for 36 upregulated contigs; **b** GO terms with a molecular function role enriched for 22 upregulated contigs; **c** GO terms with a biological process role enriched for 41 downregulated contigs; **d** GO terms with a molecular function role enriched for 29 downregulated contigs

Results from the functional annotation enrichment analyses obtained using GOseq were summarized and presented in Figs. 2, 3, 4 and 5, a list of contigs associated with the enriched Gene Ontology (GO) terms is provided in Additional file 2. In addition, the 10 most significantly enriched Protein family (Pfam) terms are presented in Table 2.

**Table 2:** Most enriched Protein families for upregulated and downregulated contigs

Upregulated			
Treatment	ID	Description	*P* value
RF Co-Pp	PF05131.9	Pep3/Vps18/deep orange family	4.58E-06
RF Co-Pp	PF10433.4	Mono-functional DNA-alkylating methyl methanesulfonate N-term	1.13E-04
RF Co-Pp	PF07650.12	KH domain	9.43E-04
RF Co-Pp	PF02798.15	Glutathione S-transferase	9.85E-04
RF Co-Pp	PF00043.20	Glutathione S-transferase	1.03E-03
RF Co-Pp	PF13410.1	Glutathione S-transferase	1.05E-03
RF Co-Pp	PF07452.7	CHRD domain	1.35E-03
RF Co-Pp	PF13409.1	Glutathione S-transferase	1.35E-03
RF Co-Pp	PF02536.9	mTERF	1.64E-03
RF Co-Pp	PF13417.1	Glutathione S-transferase	1.74E-03
SF Co-Pp	PF05368.8	NmrA-like family	9.92E-07
SF Co-Pp	PF13460.1	NADH(P)-binding	2.18E-06
SF Co-Pp	PF00067.17	Cytochrome P450	2.45E-06
SF Co-Pp	PF01073.14	3-beta hydroxysteroid dehydrogenase/isomerase family	4.78E-06
SF Co-Pp	PF01370.16	NAD dependent epimerase/dehydratase family	9.43E-06
SF Co-Pp	PF13504.1	Leucine rich repeat	1.04E-05
SF Co-Pp	PF00560.28	Leucine Rich Repeat	2.60E-05
SF Co-Pp	PF13855.1	Leucine rich repeat	3.79E-05
SF Co-Pp	PF13854.1	Kelch motif	4.60E-05
SF Co-Pp	PF13516.1	Leucine Rich repeat	4.96E-05
RF Co-PpPc	PF13229.1	Right handed beta helix region	2.73E-10
RF Co-PpPc	PF14368.1	Probable lipid transfer	3.90E-09
RF Co-PpPc	PF00230.15	Major intrinsic protein	4.07E-09
RF Co-PpPc	PF00234.17	Protease inhibitor/seed storage/LTP family	9.65E-09
RF Co-PpPc	PF00657.17	GDSL-like Lipase/Acylhydrolase	2.17E-08
RF Co-PpPc	PF07731.9	Multicopper oxidase	2.48E-08
RF Co-PpPc	PF12708.2	Pectate lyase superfamily protein	2.36E-07
RF Co-PpPc	PF00759.14	Glycosyl hydrolase family 9	2.54E-07
RF Co-PpPc	PF00394.17	Multicopper oxidase	3.01E-07
RF Co-PpPc	PF07732.10	Multicopper oxidase	4.23E-07
SF Co-PpPc	PF07042.6	TrfA protein	9.92E-04
SF Co-PpPc	PF13222.1	Protein of unknown function (DUF4030)	1.07E-03
SF Co-PpPc	PF03511.9	Fanconi anaemia group A protein	1.68E-03
SF Co-PpPc	PF07963.7	Prokaryotic N-terminal methylation motif	1.77E-03
SF Co-PpPc	PF08412.5	Ion transport protein N-terminal	1.77E-03
SF Co-PpPc	PF02041.11	Auxin binding protein	2.55E-03
SF Co-PpPc	PF02578.10	Multi-copper polyphenol oxidoreductase laccase	2.63E-03
SF Co-PpPc	PF12076.3	WAX2 C-terminal domain	2.86E-03
SF Co-PpPc	PF07393.6	Exocyst complex component Sec10	3.02E-03
SF Co-PpPc	PF02522.9	Aminoglycoside 3-N-acetyltransferase	3.05E-03
Downregulated			
RF Co-Pp	PF14225.1	Cell morphogenesis C-terminal	1.50E-04
RF Co-Pp	PF05004.8	Interferon-related developmental regulator (IFRD)	1.85E-04
RF Co-Pp	PF08167.7	rRNA processing/ribosome biogenesis	4.64E-04
RF Co-Pp	PF06146.7	Phosphate-starvation-inducible E	9.10E-04
RF Co-Pp	PF12348.3	CLASP N terminal	1.04E-03
RF Co-Pp	PF13798.1	Protein of unknown function with PCYCGC motif	1.54E-03
RF Co-Pp	PF10248.4	Myelodysplasia-myeloid leukemia factor 1-interacting protein	2.43E-03
RF Co-Pp	PF11305.3	Protein of unknown function (DUF3107)	2.47E-03
RF Co-Pp	PF14151.1	YfhD-like protein	2.96E-03
RF Co-Pp	PF01690.12	Potato leaf roll virus readthrough protein	3.02E-03
SF Co-Pp	PF07732.10	Multicopper oxidase	1.50E-14
SF Co-Pp	PF00394.17	Multicopper oxidase	1.48E-13
SF Co-Pp	PF07731.9	Multicopper oxidase	4.20E-13
SF Co-Pp	PF00225.18	Kinesin motor domain	2.17E-11
SF Co-Pp	PF00759.14	Glycosyl hydrolase family 9	2.07E-08
SF Co-Pp	PF06525.6	Sulfocyanin (SoxE)	3.46E-08
SF Co-Pp	PF00230.15	Major intrinsic protein	1.74E-07
SF Co-Pp	PF00091.20	Tubulin/FtsZ family	2.03E-07
SF Co-Pp	PF13229.1	Right handed beta helix region	4.66E-07
SF Co-Pp	PF12708.2	Pectate lyase superfamily protein	5.02E-07
RF Co-PpPc	PF03055.10	Retinal pigment epithelial membrane protein	1.44E-07
RF Co-PpPc	PF00332.13	Glycosyl hydrolases family 17	2.84E-06
RF Co-PpPc	PF01738.13	Dienelactone hydrolase family	1.73E-05
RF Co-PpPc	PF00670.16	S-adenosyl-L-homocysteine hydrolase	3.31E-05
RF Co-PpPc	PF04101.11	Glycosyltransferase family 28 C-terminal domain	5.33E-05
RF Co-PpPc	PF01973.13	Protein of unknown function DUF115	6.11E-05
RF Co-PpPc	PF13528.1	Glycosyl transferase family 1	1.02E-04
RF Co-PpPc	PF00300.17	Histidine phosphatase superfamily (branch 1)	1.80E-04
RF Co-PpPc	PF02772.11	S-adenosylmethionine synthetase	2.47E-04
RF Co-PpPc	PF00221.14	Aromatic amino acid lyase	3.04E-04
SF Co-PpPc	PF10604.4	Polyketide cyclase / dehydrase and lipid transport	2.47E-04
SF Co-PpPc	PF03169.10	OPT oligopeptide transporter protein	5.82E-04
SF Co-PpPc	PF00190.17	Cupin	6.22E-04
SF Co-PpPc	PF00407.14	Pathogenesis-related protein Bet VI family	8.53E-04
SF Co-PpPc	PF05360.9	yia A/B two helix domain	2.28E-03
SF Co-PpPc	PF03547.13	Membrane transport protein	3.25E-03
SF Co-PpPc	PF02442.12	Lipid membrane protein of large eukaryotic DNA viruses	3.58E-03
SF Co-PpPc	PF07963.7	Prokaryotic N-terminal methylation motif	3.80E-03
SF Co-PpPc	PF05153.10	Family of unknown function (DUF706)	3.99E-03
SF Co-PpPc	PF01092.14	Ribosomal protein S6e	4.35E-03

Table shows the top ten most enriched Protein families for up- and downregulated contigs in leaves harvested from oak microcuttings during root (RF) and shoot flush growth stages (SF) treated with *P. penetrans* (Co-Pp) and the co-inoculation of *P. penetrans* and *P. croceum* (Co-PpPc), Protein family (Pfam) ID, Pfam term description and significance level (*P*-value) are provided

### *Effects of* Pratylenchus penetrans *at root flush*

Gene expression response in leaf tissue indicated oxidative stress and plant defence elicitation during RF. GO terms involved in hypersensitive response such as *lignin catabolism* or detoxification of Reactive oxygen species (ROS) such as *superoxide metabolism*, *hydroquinone oxygen oxidoreductase* and *lactoglutathione lyase activity* (Fig. 2a, b) and the Pfam term *glutathione*-*S*-*transferase* (Table 2) were enriched in upregulated contigs (EUC).

The EUC GO terms *allene*-*oxide cyclase activity* a precursor of jasmonic acid biosynthesis and *sulphur compound biosynthesis* and *2*-*C*-*methyl*-*D*-*erythritol 4*-*phosphate cytidyltransferase activity* (Fig. 2a, b) indicate induction of the Jasmonic acid pathway and production of secondary metabolites participating in defence, respectively. Meanwhile, GO terms enriched in downregulated contigs (EDC) *cellular response to glucose starvation* and *protein kinase* (Fig. 2c, d), show a repression of pathogen perception.

Apart from plant defence activation processes related to plant growth were promoted in response to *P. penetrans* indicated by EUC GO terms *positive regulation of cell proliferation* and *lipid metabolism* (Fig. 2 a), and Pfam term *Chordin* (*CHRD*) *protein* which participates in regulation of basic and vital cellular processes (Table 2) during RF.

### *Effects of* Pratylenchus penetrans *at shoot flush*

Systemic transcriptomic response in microcutting leaf tissue to *P. penetrans* was distinctly stronger during SF (Fig. 1). There was positive regulation of genes related to plant defence, pathogen resistance and metabolism of secondary compounds with defence properties shown by EUC GO terms *steroid* and *flavonoid biosynthesis*, *leucocyanidin oxygenase activity*, *naringenin*-*chalcone synthase activity*, *terpene synthase activity* and *chitinase* activity (Fig. 3a, b). Correspondingly, EUC Pfam terms *3*-*beta hydroxysteroid dehydrogenase family* and *Cytochrome P450* both involved in plant defence and *leucine rich repeats* (*LRR*) *proteins* were among the top enriched Pfam terms (Table 2). On the other hand EDC GO terms *pectinesterase inhibitor* as well as *signal transducer activity* (Fig. 3d) suggest a dampening of plant signal transduction.

During SF carbon metabolism was altered in response to *P. penetrans*, EUC GO terms *regulation of photosynthesis*, *photosynthesis acclimation*, glucose-6-phosphate transport and *glucose*-*6*-*phosphate transmembrane transporter activity* (Fig. 3a, b) and Pfam term *Nicotinamide adenine dinucleotide* (*NAD*) *dependent epimerase*/*dehydratase family* (Table 2) indicate changes in glucose translocation. Moreover, GO terms *sucrose metabolism* and *carbohydrate metabolism* were EDC (Fig. 3c), in sum pointing to an accumulation of glucose in aboveground tissue.

Cell growth processes were hampered in oak leaf tissues in response to the nematode during SF, EDC GO terms *microtubule motor activity*, *movement* and *binding*, *regulation of DNA replication*, *cytokinesis by cell plate formation* and *cell proliferation* (Fig. 3c, d) and Pfam terms *kinesin motor domain* and *tubulin family proteins* (Table 2). Further, cell wall formation and organisation was repressed indicated by EDC GO terms *cellulose synthase activity*, *lignin catabolism* and *pectate lyase* (Fig. 3c, d) and Pfam terms *glycosyl hydrolase family 9*, *right*-*handed beta helix region* and *pectate lyase* (Table 2).

### *Interaction with* Pratylenchus penetrans *and* Piloderma croceum *during root flush*

Oak systemic transcriptomic pattern was greatly modified in response to the co-inoculation with *P. penetrans* and *P. croceum* (Fig. 4) compared to singular inoculation with *P. penetrans* (Fig. 2) during RF, predominantly plant defence was differentially regulated. Pathogen perception signalling and plant resistance was elicited the GO terms EUC included *response to biotic stimulus*, *phosphorylation of inositol*, *wax* and *cutin biosynthesis* and polyamine metabolism (enzymes related to spermidine or spermine) (Fig. 4a, b) and correspondingly Pfam terms *Multicopper oxidase* and *GDSL*-*like lipase*/*acylhydrolase* (Table 2). On the other hand plant defence was repressed; EDC GO terms *killing cells of other organisms*, *phenylpropanoid metabolism and shikimate dehydrogenase activity*, along with *flavonoid and ethylene biosynthetic process* (Fig. 4c, d). Further reflecting this, Pfams terms *aromatic amino acid lyase* a phenylpropanoid biosynthesis catalyst and *S*-*adenosylmethionine synthase* a precursor of ethylene and polyamines were EDC (Table 2).

Plant primary metabolism was altered in response to inoculation of *P. penetrans* with *P. croceum*; GO terms EDC including *cellular*, *carbohydrate* and *amine metabolism* as well as *glycolytic process* (Fig. 4c). In contrast, the terms EUC *lipid metabolism* (Fig. 4a) and Pfam term *probable lipid transfer* (Table 2) supporting the above findings of increased signalling processing.

The systemic transcriptomic pattern shows that growth processes were promoted during RF, the GO terms EUC include *regulation of meristem growth*, *cell wall organization*, *cell replication* as well as *cellulase* and *pectate lyase activity* (Fig. 4 a, b). Additionally, EUC terms *anaphase*, *microtubule based movement* and *cytokinesis by cell plate formation* (Fig. 4 a) and Pfams terms *glycosyl hydrolase family 9* and *pectate lyase superfamily protein* (Table 2) indicates promotion of cell replication processes in microcutting leaves in response to co-inoculation with *P. penetrans* and *P. croceum*.

### *Interaction of* Pratylenchus penetrans *and* Piloderma croceum *during shoot flush*

The systemic response of oak microcuttings to co-inoculation of *P. penetrans* with *P. croceum* (Fig. 5) was distinctly lower and portrayed a different response pattern in comparison to the singular *P. penetrans* treatment (Fig. 3). Signal transduction was activated EUC GO term *intracellular cyclic nucleotide activated cation channel activity* (Fig. 5b), however hypersensitive response was repressed, EDC GO terms regulation of *phosphoprotein phosphatase activity*, *NADH pyrophosphatase* and *protein phosphatase inhibitor* (Fig. 5c, d) and correspondingly Pfam terms *pathogenesis*-*related protein Bet VI family* and *polyketide cyclase*/*dehydrase* (Table 2). The EUC GO terms *asparagine biosynthesis* and *cellular response to sucrose starvation* (Fig. 5c) are involved in metabolic alterations facilitating cell death during plant-pathogen interactions. Further, EUC GO terms *cysteine metabolism*, *selenium compound metabolism*, *response to selenium ion* and *superoxide dismutase* (Fig. 5a, b) indicate enhanced activation of antioxidative defence showing that presence of *P. croceum* dampened host defences. On the contrary, wound inducible plant defence was elicited EUC GO terms *aldehyde catabolism*, *quinone binding*, *alkane biosynthesis*, *aldehyde decarbonylase activity* and *triglyceride lipase activity* (Fig. 5a) and Pfam terms EUC *multi*-*copper polyphenol oxidoreductase laccase* and *wax 2 C*-*terminal domain proteins* (Table 2).

Plant secretion and transport of proteins was modified, GO terms *protein retention in Golgi apparatus* and *dolichyl*-*phosphate beta*-*glucosyltransferase activity* (Fig. 5a, b) and Pfam *Exocyst complex 3 component Sec10* (Table 2) were EUC. In addition, EDC included *L*-*asparagine biosynthesis*, *asparagine synthase*, *transferase activity* and *oligopeptide transporter activity* (Fig. 5c, d) and corresponding Pfams *ribosomal protein S6* and *OPT oligopeptide transporter protein* (Table 2).

Cell growth processes were altered in response to co-inoculation of *P. penetrans* and *P. croceum*, and in contrast to during RF, processes related to cell replication or expansions were inhibited indicated by GO terms *cell plate formation*, *microtubule organization* and *syncytium formation* (Fig. 5d). Lateral root growth process was favoured over apical growth indicated by GO terms EUC *root epidermal cell differentiation*, *Rho GDP*-*dissociation inhibitor activity* (Fig. 5 a, b) and EDC term *high affinity potassium ion import* (Fig. 5 c). Correspondingly linked to control of growth and development processes was EUC Pfam term *Auxin binding protein* while the auxin efflux carrier *Membrane transport protein* (Table 2) was EDC.

## Discussion

### Systemic oak response induced by plant parasitic nematode

Oak microcuttings systemic transcriptomic responses showed that wide ranges of defence mechanisms were employed against *P. penetrans* infection indicating that plant basal immunity was activated [30, 31]. Plants have complex multi-layered defence mechanisms, involving the recognition of pathogen perception and subsequent activation of various protection strategies that suppress infection locally or prime distant tissues via systemic defence signalling [32, 33]. Such transcriptional defence responses in plant-nematode interactions are well recognized for annual agricultural plants [26, 34, 35] and the present study shows that plant-parasitic nematodes induced a comparable response in oak, a perennial tree. With regards to migratory nematodes, like in the present study, induction of pathogen-triggered immunity remains persistent regardless of the time point after inoculation [20, 36]. Accordingly, across the oak growth stages, the interaction with the plant-parasitic nematode induced systemic transcriptomic responses in leaf tissue. These can be categorized into three major processes: i) elicitation of plant defence, ii) repression of host defence, and iii) modulation of carbon metabolism.

Oak defence elicitation by nematodes was demonstrated by the upregulation of disease resistance genes encoding for proteins of the Toll interleukin-1 receptor (TIR)- nucleotide binding site (NBS) - LRR, nucleotide binding (NB)- APAF-1, R proteins, and CED-4 (ARC) and LRR classes. Comparably, in tomato plants *Mi*-*1* gene containing an LRR region plays a role in signalling processes that confer resistance to the root-knot nematode [37, 38]. Hormonal signalling pathways were activated, particularly induction of genes related to the biosynthesis of salicylic acid, jasmonic acid and ethylene. These plant hormones are well known to govern systemic induced defence responses against pathogens [39, 40], and their induction was shown for migratory endoparasitic nematodes in rice [36, 41]. Additionally, proteins involved in the shikimate pathway as well as the biosynthesis of steroids and flavonoids were upregulated, which corresponds to reports on the increase of glucosinolates, phenolics or terpenoids in systemic tissues after nematode infection of plant roots [42–44]. In sum these molecular patterns indicate a strong systemic defence response of oaks to *P. penetrans*. Such defence compounds have been shown to play an important role belowground, flavonoids alter the motility and hatching of nematodes [45]. However, the distinct and diverse changes in metabolic profiles of leaf tissues suggest a priming effect of aboveground tissues of oak. Previous studies have shown that belowground feeding by *P. penetrans* induced systemic resistance against insect herbivores in host leaves [46, 47].

*P. penetrans* triggered production of ROS in the microcuttings leaf tissue, this has been reported in roots of *A. thaliana* and tomato infected with *H. glycines* and *M. incognita* [24, 48]. This oxidative burst kills any organism in contact with the superoxide radicals and causes plant cell death in different plant-pathogen systems moreover it drives cross-linking of structural proteins reinforcing the cell wall as a physical [49–51]. ROS has also previously been linked to the facilitation of biotrophic interactions by suppression of plant cell death [52, 53]. However, ROS also plays a signalling role mediating defence genes activation following pathogen infection [54, 55]. It is likely that the enhanced production of ROS in oak indicates a signalling role over long distances, leading to priming different plant tissues [56]. In addition, the abundant EUC Pfam term *Glutathione*-*S*-*transferase* (Table 2) indicates increased ROS homeostasis further supporting the role of ROS in signaling cascades in oak leaf tissues in response to *P. penetrans* infection. The role of ROS as signaling molecules is possible when non-toxic levels of ROS are present in cells preventing cell death, therefore, a balance between production and the metabolic counter-process pathways must be maintained hence the increased activity of *Glutathione*-*S*-*transferase* enzymes [57, 58].

To repress host defence, plant-parasitic nematodes including *Pratylenchus spp*. were shown to secrete immune-modulatory effectors that hijack host signalling pathways to aide parasitism [14, 59, 60]. In the present study, enzymes involved in the synthesis of signalling molecules, expressed in response to wounding *probable cytidinediphospho* (*CDP*) -*diacylglycerol*-*inositol 3 phosphatidyltransferase 2* and *inositol 1*,*3*,*4*-*trisphosphate 5*/*6*-*kinase family protein* (Additional file 2) were downregulated in microcuttings leave tissues in response to *P. penetrans* infection. These glycerolipids molecules play a key role in immune response signalling and mediate plant defence responses to herbivory [33, 61, 62]. Plants defective in the production of myo-inositol, a building block for these secondary messengers, are more susceptible to pathogen infections [63]. Moreover, a recent study by Kyndt et al. [20] found that the suppression of defence systemically by root-knot nematodes potentially makes rice plants more vulnerable to aboveground pathogen attack.

The nematode additionally altered the carbon metabolism in microcuttings, mediated by three predominant processes. Firstly, pathogen attacks are often connected to the levels of sugar in plant cells, for instance glucose activates expression of resistance genes while sucrose functions as a signalling molecule [64–66]. During the infection with *P. penetrans* sucrose non-fermenting-1 related protein kinase (SnRK1), GO term *cellular response to glucose starvation* a key metabolic regulator altering defence mechanisms against biotic and abiotic stress [67] was repressed. Secondly, transcripts encoding for enzymes such as cellulose synthase, raffinose synthase, sucrose synthases and beta-galactosidases were downregulated pointing to an accumulation of glucose in oak leaves. This altering source and sink metabolism in plants is likely a response to stress as shown by Ehness et al. [68]. Thirdly, *P. penetrans* induced genes enriched for GO terms *regulation of photosynthesis* and *photosynthesis acclimation*, while transcripts encoding *photosystem II light harvesting complex B1B2* and *high chlorophyll fluorescence 243* were downregulated. This is indicative of repressed photosynthesis and has been reported in incompatible plant-pathogen interactions where plants switch off photosynthesis and other carbon dependent metabolic pathways to initiate processes required for respiration and defence [69, 70]. Studies on plant-nematode interactions revealed reduction of carbon fixation in coffee by *Pratylenchus coffeae*, photosynthesis in tomato by *M. javanica*, and the amount of chlorophyll in systemic tissues of rice by *H. oryzae* [41, 71, 72]. In sum, these alterations in oak photosynthesis and carbon metabolism induced by *P. penetrans* are likely to be part of the plants strategy in enhancing defence referred to as “to gain fuel for the fire” by Bolton [73].

### Effect of plant growth stage on biotic interactions

The systemic response of oak to *P. penetrans* was greatly influenced by the plant’s developmental stage. The systemic response in oak to the nematode was much stronger during SF, demonstrated by the 16-fold increase of differentially expressed genes as compared to during RF. This finding is consistent with Kurth et al. [74], using the same model microcosm system observed a larger systemic response expression in oak microcuttings to a mycorrhiza helper bacterium *Streptomyces* sp. AcH505 during SF.

The observed weak response of oaks to *P. penetrans* during RF likely mirrors the endogenous nutrient allocation pattern of oaks. Generally, plant parasitic nematodes create a carbon and nutrient sink to roots [75], RF coincides with a strong flow of carbohydrates belowground [4, 5] whereby nematode infection during this stage did not increase the root sink strength to an extent that provoked a strong defence reaction. Instead, cell proliferation was positively regulated with contigs encoding for proteins involved in nucleic binding, regulation of transcription, promoting replication, cell plate formation induced during RF. In addition, lipid metabolism and lignin catabolism were activated, with the former being vital for membrane biogenesis and the latter for plant growth [76]. Together these processes indicate cell generation processes elicited in response to *P. penetrans*, which may point to compensatory plant growth, as reported in response to nematode infection in crops and grass [77, 78]. However, enhanced growth was not confirmed by biomass data, as after 10 days plant-nematode interaction no effect on oak growth pattern was apparent (Additional file 3).

Meanwhile, during SF when oaks allocate carbon predominantly in aboveground tissues, the feeding by *P. penetrans* induced a diverse pattern of defence mechanisms. Most likely this distinct plant response results from the strong carbon demand of the nematode imposed at a growth stage where oaks retain photoassimilates in shoots [4, 5]. This allocation of sugars in sink leaves was further enhanced in response to *P. penetrans* indicated by repressed genes involved in carbon metabolism as well as enzyme activities of cellulose, raffinose and sucrose synthases. By reallocation of resources away from the site of attack, here the oak roots, plants may safeguard them for future growth or to synthesize defensive secondary metabolites, both well-known strategies under herbivore attack [79]. Such enhanced elicitation of plant defence through activation of pathways such as phenylpropanoid and isoprenoid producing defence metabolites as well as PR-proteins and callose deposition point to a major flow of carbon from primary into secondary metabolism [73].

In summary the response of oaks to *P. penetrans* was greatly modulated by the plant growth stage. During RF the plant-parasite relationship apparently was quite balanced, whereas during SF *P. penetrans* triggered a strong systemic defence response and alteration of primary metabolism including transcriptional reprogramming of photosynthesis and physiological mechanisms. This stage dependent huge difference in the transcriptomic profiles in oak systemic tissues clearly shows that the endogenous rhythmic growth resource-linked allocation pattern determines host response to pathogens; therefore, it should be considered in future investigations.

### *Effects of interaction of* P. penetrans *and* P. croceum

The transcriptomic response to *P. penetrans* in oaks was modified by the presence of the ectomycorrhizal fungus *P. croceum* at both growth stages. Strikingly, the magnitude of genes expressed was vice versa, high and low during RF and SF, respectively. The interaction with the mycorrhizal fungus apparently changed the susceptibility of microcuttings to the plant-parasitic nematode resulting in complete reprogramming of host response.

During RF the presence of *P. croceum* enhanced pathogen perception in oak demonstrated by EUC *response to biotic stimulus*. However host defence was suppressed by *P. croceum*, *pathogenesis related gene 5* and *thaumatin superfamily proteins* were downregulated (Additional file 2), while proteins interfering with salicylic acid-regulated pathogenesis resistance such as *auxin induced proteins* [80] were upregulated. Numerous plant defence mechanisms such as *killing cells of other organisms* and *flavonoid*, *ethylene* and *phenylpropanoid metabolism* were repressed. Overall, this gene expression pattern points to impaired plant resistance and defence, and is in line with Caravaca et al. [81] who showed that, compared to SF oak microcuttings inoculated with the ectomycorrhizal fungus *P. croceum* were more susceptible to *P. penetrans* during RF. This is supported by the downregulation of primary metabolism i.e. cellular, amine, carbohydrate and glycolysis, indicating a negative impact on plant performance. Our finding suggests a plant strategy of retaining nutrient reserves to impair the performance of these root invaders since both the nematode and the fungus draw carbon from the oak host increasing the sink strength of roots likely offsetting the equilibrium observed in singular *P. penetrans* treatment.

Similarly during SF the presence of *P. croceum* modulated the response pattern of oak to *P. penetrans* infection; however, there is a striking dissimilarity with the singular *P. penetrans* treatment. The very low number of DECs shows that *P. croceum* strongly supressed oak’s response to the pathogen during SF, this finding is consistent with Kurth et al. [74] who reported similar effects for the interaction of oak with *P. croceum* and the mycorrhiza helper bacterium AcH 505. The downregulation of GO terms EDC such as *NADH pyrophosphatase* and *protein phosphatase inhibitor* involved in triggering hypersensitive response and accumulation of pathogenesis related proteins indicate a suppression of oak defence responses. Similar findings of host defence suppression by ectomycorrhizal fungi have been reported for the host trees *Quercus suber* and *Populus Sp*. and their respective symbiotic fungi *Pisolithus tinctorius* and *Laccaria bicolor* [28, 82]. Furthermore, our study found that vesicle-mediated trafficking was altered, in particular secretion and transport of proteins in the presence of *P. croceum*, a well-known strategy applied by fungi to evade plant defences [29, 83].

## Conclusions

Plant parasitic nematodes caused multi-layered transcriptomic changes in the physiology and metabolism of pedunculate oak. *P. penetrans* differentially regulated plant genes related to defence response, changes in cell wall architecture and altered carbon allocation compared to the control. However, this transcriptomic pattern was distinctly shaped by oak endogenous rhythm and, moreover, altered by the presence of the mycorrhizal symbiont *P. croceum*. During RF when carbon is primarily channelled belowground, the defence against the nematode solely was subtle compared to its co-inoculation with *P. croceum*. Both biotic interactors likely increase sink strength of roots, resulting in a distinct oak defence answer. In contrast, during SF the joint interaction with *P. croceum* led to suppression of the oak response to nematodes. Furthermore, oak primary metabolism was altered particularly genes involved in photosynthesis and metabolism, likely a strategy by plants to reallocate nutritional reserves predominantly aboveground. In sum, the outcome of the interplay between root herbivores and oak was considerably driven by the plant’s endogenous rhythmic growth. This indicates that differences in life strategy, i.e. resource allocation related to endogenous growth in perennial trees versus annual plants, influences costs and benefits investment in plant defence and should be taken into account in future investigations.

## Methods

### Oak microcutting culture system

We used pedunculate oak microcuttings propagated from the clone DF159 (*Quercus robur*) and rooted as described by [84]. Microcuttings were grown in soil-based microcosms, 12 × 12 cm petri dishes filled with γ-sterilized soil collected from an oak forest stand as described in detail by Herrmann et al. [27]. Half of the microcuttings were inoculated with the ectomycorrhizal fungus *Piloderma croceum* (J. Erikss and Hjortst). In brief, an inoculum of *P. croceum* (strain F1598) was pre-cultured on modified Melin-Norkrans medium [85]. A solid inoculum was produced in a substrate mixture of vermiculite and sphagnum peat and incubated at 20 °C for 4 weeks in the dark. Petri dishes were filled with a thoroughly mixed soil medium made up of equal volumes 1:1 (v/v) of the γ-sterilized soil and *P. croceum* inoculum substrate. Five weeks after the establishment of the oak microcuttings microcosms, 5 ml of a diluted (1/1000) bacterial filtrate [86] was added to each mesocosm, whether inoculated with mycorrhizal or not, to re-establish a natural microbial community.

The oak microcuttings were cultured in a climate chamber at 23 °C, 16:8 h day: night regime, with photon flux density of 180 μmolm^−2^s^−1^, 400 ppm CO_2_ and 80 % relative humidity. Plant development was recorded bi-weekly using four stages to characterize each growth cycle: bud rest (A), bud swelling (B), shoot elongation (C) and leaf expansion (D) [3]. Owing to the characteristic endogenous rhythmic growth pattern of oak, in the performed analyses the stage B corresponding with maximal root elongation represented the root flush (RF) and the stage D corresponding with maximal leaf expansion represented the shoot flush (SF) [4].

### Root herbivore nematodes

A generalist cosmopolitan invertebrate soil nematode, *Pratylenchus penetrans* (Cobb), was used as belowground root herbivore model. Ethics approval was not required for any aspect of this study; animal research legislation does not apply to soil nematodes.

Axenic cultures of *P. penetrans* were grown and multiplied on carrot discs following the protocol by O’Bannon and Taylor [87]. Nematodes were extracted from carrot discs using the Baermann method [88] over a period of 48 h at room temperature. The extracted nematodes were surface sterilized by soaking in 0.01 % mercury chloride solution for 10 min and washed in autoclaved Volvic water, with the washing step repeated three times. Nematode density was achieved by counting individuals in a known volume of water, which was then adjusted to obtain the desired nematode inoculum density per ml.

### Experimental design

Eight weeks after establishment of oak microcuttings in the microcosm, plants were randomly assigned to four treatments with 10 replicates each in a full factorial experimental design: Control - no fungus or nematodes (Co), *P. croceum* (Pc), *P. penetrans* (Pp) and co-inoculation of *P. penetrans* and *P. croceum* (PpPc). Half of the plants previously inoculated with or without the ectomycorrhiza fungus *P. croceum*, were inoculated with *P. penetrans* at a rate of 2,300 nematodes per plant, done by inserting a 1 ml pipette tip adjacent to microcuttings root system and releasing the nematode suspension aliquots.

Ten days post nematode inoculation; oak microcuttings sorted according to their development stages at RF or SF served for harvest. The terminal developed leaves from plants at RF (source leaves) and the just formed young leaves at SF (sink leaves) harvested from individual microcuttings for each treatment were weighed, wrapped in aluminium foil, and immediately submerged in liquid nitrogen and stored at -80 °C. To check for infection of the microcuttings by *P. penetrans*, an additional three plants were harvested per treatment and their roots were stained with acid fuchsin 10 days after infection. Roots were boiled for 3 min in 0.8 acetic acid and 0.013 % acid fuchsin, washed with running tap water and then destained in acid glycerol. Roots were checked for presence of *P. penetrans* using a stereomicroscope at 50x magnification.

### RNA assays

For the systemic transcriptomic analyses leaf samples derived from 3–4 microcuttings at the same developmental stage per treatment were pooled to provide sufficient material for RNA extraction. Three RF and two SF biological replicates were obtained for each treatment. RNA was extracted using the MasterPure Plant RNA Purification Kit (Epicentre, Germany). RNA integrity and quantification was performed using gel electrophoresis, a Nanodrop1000 spectrophotometer (Thermo Scientific, Waltham, MA, USA) and Bioanalyzer 2100 (Agilent). RNA sequencing was performed at the Beijing Genomics Institute (Hong Kong, China). Briefly, 100 bp paired-end Illumina Truseq version 2 libraries were constructed and sequenced using the Illumina HiSeq2000 sequencing platform. The sequence data was deposited as fastq files to the NCBI Short Read Archive linked to a report specific BioProject termed PRJNA330761.

### Read processing and analysis of differential expression

The Illumina sequenced data set was processed according to Tarkka et al. [9]. Bioinformatics software SeqClean (ftp://occams.dfci.harvard.edu/pub/bio/tgi/software/) that uses custom Java scripts was used to remove all low quality nucleotides (quality score < 20), poly-A-tails and sequences shorter than 50 bp. The processed Illumina reads were then mapped to the OakContigDF159.1 reference transcriptome [9] using BOWTIE, an alignment program [89]. Software tool RSEM was used for quantification of transcript abundances [90]. Negative binomial models were fitted to the transcript abundances determined by RSEM and the fold-change was calculated by pairwise comparisons using the edgeR function [91] of the Bioconductor package [92] in R (R core group, http://www.r-project.org/). Benjamini-Hochberg false discovery rate (FDR) was performed to adjust *P*-values; significance for differential expression during pairwise comparison was set at FDR < 0.01.

The description of individual contigs was made using Blast2GO based on up to 20 hits against the NCBI NR - database (E-value 1e-5). Homologues for oak contigs were determined by performing a BLASTX search against *Arabidopsis thaliana* L. TAIR online database [93]; only hits with an E-value of at least 1e-5 were considered for the assignment.

Functional analysis of the differentially regulated genes to make efficient biological inferences was performed using the Gene Ontology and Protein family enrichment analysis methods. Bioconductor software package GOseq, which is capable of overcoming the length bias due to over-detection of differential expression from long and highly expressed transcripts inherent to RNA-Seq data [94] was used for these analyses. GOseq performs a statistical test based on a hypergeometric distribution to determine if in a given list of DE tags (e.g. genes or contigs) tags assigned to a certain category (e.g. GO terms) are significantly enriched, i.e. if they occur more frequently than expected by chance. Thereby GOseq adjusts the estimation of the *P*-value for tag-length; a *P*-value < 0.05 was considered as significant. GO is a hierarchically organized collection of functional gene sets based on a controlled vocabulary that classifies gene products at protein domains by biological process, molecular function and cellular component [95]. Enriched GO terms were condensed and visualized using REVIGO [96]. The OakContigDF159.1 reference library, GO annotations as well as best blast hits of each contig have been deposited at www.trophinoak.de.

## Abbreviations

ARC, APAF-1, R proteins, and CED-4; CDP, Cytidinediphospho; enriched in upregulated contigs; DE, differentially expressed; DECs, differentially expressed contigs; EDC, enriched in downregulated contigs; EUC, CHRD, plant Chordin protein; FC, fold of change; FDR, false discovery rate; GO, Gene Ontology; LRR, leucine rich repeats; NAD, nicotinamide adeninde dinucleotide; NB, nucleotide binding; Pc, *Piloderma croceum*; Pfam, Protein family; Pp, *Pratylenchus penetrans*; PpPc, co-inoculation of P. *penetrans* and *P. croceum*; RF, root flush; ROS, reactive oxygen species; SF, shoot flush; SnRK1, sucrose non-fermenting 1-related protein kinase; TIR, toll, interleukin-1, and R proteins

## Additional files

Additional file 1: Title: Common differentially expressed contigs between treatments. Description: List of common differentially expressed contigs and the respective differential expression level (Log_2_ of FC) determined by edgeR with a threshold Benjamini-Hochberg adjusted *P* < 0.01 as cut-off following pairwise comparison of Control versus *P. penetrans* (Co-Pp) and Control versus the co-inoculation of *P. penetrans* with *P. croceum* (Co-PpPc) during root and shoot flush. (XLSX 12 kb) Additional file 2: Title: Differentially expressed contigs associated with the enriched GO terms presented in Figs. 2, 3, 4 and 5. Description: List of DECs associated with enriched GO terms, the respective gene description and the significant differential expression (Log_2_ of FC) determined by edgeR with a threshold Benjamini-Hochberg adjusted *P* < 0.01 as cut-off indicated by FDR. Control versus *P. penetrans* treatment during root flush (RF Co-Pp), Control versus *P. penetrans* treatment during shoot flush (SF Co-Pp), Control versus co-inoculation of *P. penetrans* and *P. croceum* treatment during root flush (RF Co-PpPc), Control versus co-inoculation of *P. penetrans* and *P. croceum* during shoot flush (SF Co-PpPc). (XLSX 79 kb) Additional file 3: Title: Dry weight of plant tissues. Description: Table of the dry weight of plant tissues at different growth stages (Root and Shoot flushes) for the respective treatments: control, *P. penetrans*, *P. croceum* and co-inoculation of *P. penetrans* and *P. croceum*. ANOVA with *, ** and *** with *P* < 0.05, 0.01 and 0.001 respectively. Data with the same or no letters are not significantly different according to Tukey HSD at *P* < 0.05. (DOCX 15 kb)
