# Supplementary material for: Transcriptome analysis in oak uncovers a strong impact of endogenous rhythmic growth on the interaction with plant-parasitic nematodes
Source: BMC Genomics. 2016 Aug 12;17:627. doi: 10.1186/s12864-016-2992-8 (PMC4982138; doi:10.1186/s12864-016-2992-8)
Supplement: Additional file 3: — Title: Dry weight of plant tissues. Description: Table of the dry weight of plant tissues at different growth stages (Root and Shoot flushes) for the respective treatments: control, P. penetrans, P. croceum and co-inoculation of P. penetrans and P. croceum. ANOVA with *, ** and *** with P < 0.05, 0.01 and 0.001 respectively. Data with the same or no letters are not significantly different according to Tukey HSD at P < 0.05. (DOCX 15 kb) [file 12864_2016_2992_MOESM3_ESM.docx]

Title: **Dry weight of plant tissues**

Description: Table of the dry weight of plant tissues at different growth stages (Root and Shoot flushes) for the respective treatments: control, *P. penetrans*, *P. croceum* and co-inoculation of *P. penetrans* and *P. croceum*. ANOVA with *, ** and *** with P < 0.05, 0.01 and 0.001 respectively. Data with the same or no letters are not significantly different according to Tukey HSD at P < 0.05.

|  | Root Flush | | | | Shoot Flush | | | |  |
| --- | --- | --- | --- | --- | --- | --- | --- | --- | --- |
|  | Control | *P. penetrans* | *P. croceum* | *P. penetrans* + *P. croceum* | Control | *P. penetrans* | *P. croceum* | *P. penetrans* + *P. croceum* | ANOVA |
| Shoot (g) | 0.17 ± 0.05 | 0.16 ± 0.04 | 0.20 ± 0.07 | 0.20 ± 0.07 | 0.15 ± 0.08b | 0.15 ± 0.06b | 0.26 ± 0.06a | 0.23 ± 0.04ab | Pilo** |
| Root (g) | 0.18 ± 0.06 | 0.15 ± 0.04 | 0.22 ± 0.09 | 0.19 ± 0.07 | 0.12 ± 0.07b | 0.13 ± 0.06ab | 0.23 ± 0.07a | 0.21 ± 0.03ab | Pilo** |
| R/S Ratio | 1.15 ± 0.48 | 0.90 ± 0.19 | 1.10 ± 0.29 | 1.03 ± 0.45 | 0.84 ± 0.15 | 0.92 ± 0.16 | 0.89 ± 0.18 | 0.94 ± 0.17 |  |
